# The long-term effects of being bullied or a bully in adolescence on externalizing and internalizing mental health problems in adulthood

**DOI:** 10.1186/s13034-015-0075-2

**Published:** 2015-08-23

**Authors:** Johannes Foss Sigurdson, A. M. Undheim, J. L. Wallander, S. Lydersen, A. M. Sund

**Affiliations:** Faculty of Medicine, Norwegian University of Science and Technology, The Regional Centre for Child and Youth Mental Health and Child Welfare (RKBU), P.O. Box 8905, MTFS, 7491 Trondheim, Norway; University of California, Merced, CA USA; St. Olav Hospital, Trondheim, Norway

**Keywords:** Longitudinal, Being bullied, Aggressive toward others, Bully–victim, Epidemiology, Mental health problems

## Abstract

**Background:**

The aim is to examine associations between bullying involvement in adolescence and mental health problems in adulthood.

**Methods:**

Information on bullying-involvement (being bullied, bully–victim, aggressive toward others) and non-involved was collected from 2464 adolescents in Mid-Norway at mean age 13.7 and again at mean age 14.9. Information about mental health problems and psychosocial functioning was collected about 12 years later at mean age 27.2 (n = 1266).

**Results:**

All groups involved in bullying in young adolescence had adverse mental health outcomes in adulthood compared to non-involved. Those being bullied were affected especially regarding increased total sum of depressive symptoms and high levels of total, internalizing and critical symptoms, increased risk of having received help for mental health problems, and reduced functioning because of a psychiatric problem in adulthood. While those being aggressive toward others showed high levels of total and internalizing symptoms. Both those being bullied and bully–victims showed an increased risk of high levels of critical symptoms. Lastly, all groups involved in bullying on adolescence had increased risk of psychiatric hospitalization because of mental health problems.

**Conclusion:**

Involvement in bullying in adolescence is associated with later mental health problems, possibly hindering development into independent adulthood.

## Background

Being involved in bullying is common among adolescents. Prevalence rates of being victims of bullying vary globally from 6 to 35 %, and bullying others from 6 to 32 %, whereas a smaller group, from 1.6 to 13 %, has experience both as a bully and victim (“bully–victim”) [1–7]. Prevalence differences are most often attributed to variations in age of participants, time range of measurement and classification of bullying. Olweus and Limber [8] defines bullying or victimization in terms of being bullied, intimidated, or victimized when a person is exposed, repeatedly and over time, to negative actions from more powerful peers. Bullying behavior may be manifested in various ways, for example, as teasing, active exclusion from a social group, or physical assaults [9]. Studies in schools have found an association between involvement in bullying—whether as victim, perpetrator or bully–victim—and elevated mental health problems [10, 11]. Surprisingly, almost no research has addressed the effects from bullying on the transition from adolescent to early adulthood when most people move on from the educational system to work-life and are expected to begin making a life apart from their parents. Accordingly, we know little about the long-term association between bullying involvement in adolescence and mental health outcomes and broader effects on development into young adulthood. Recently a few studies have indicated troubling associations between bullying involvement and later problems in adulthood [1, 5, 6]. Nonetheless, further prospective longitudinal research on bullying involvement in adolescence and later mental health outcomes is much needed.

A common way of examining mental health issues separates those reflecting internalizing and externalizing problems. Whereas, the terms internalizing and externalizing problems have traditionally mainly been used to describe symptoms occurring in childhood, they are also applied in adult psychiatric research due to the latent structure of psychiatric disorders [12, 13]. Internalizing symptoms include problems within the individual, such as depression, anxiety, fear and withdrawal from social contacts. Some research suggests that internalizing problems are more prevalent in victims of bullying [8]. However, other research has been inconsistent [14]. A recent longitudinal study has shown that both those who are bullied and bullying others in adolescence have an increased risk of developing panic-disorder or depression in young adulthood; in addition, those being bullied had an increased risk of developing anxiety disorders [1].

Externalizing symptoms reflect behaviours that are directed outwards toward others such as anger, aggression, and conduct problems including a tendency to engage in risky and impulsive behaviour, as well as criminal behaviour. Individuals who are aggressive and bully others not surprisingly concurrently display more externalizing symptoms than those being bullied and peers who have no involvement in bullying [15]. Importantly, research suggests that bullying others in adolescence is associated with elevation in externalizing symptoms as young adults [1, 16]. Sourander et al. [16] found that being a frequent bully at age 8 predicted antisocial personality, substance abuse, and depressive and anxiety disorders in early adulthood. However, the sample consisted only of males during enrollment at the Finish obligatory military service. Copeland and colleagues [1] reported in a prospective study that those bullying others in adolescence have heightened risk of developing antisocial personality-disorder in young adulthood, even when controlling for preexisting psychiatric problems, family hardships, and child maltreatment.

In addition to concerns about psychopathology, there have been several reports of long term impairments in psychosocial functioning among those involved in bullying, including mental and physical health, school functioning, and peer relations. Aggression toward peers is associated with poor school performance and conduct problems among students 7–9 years of age [17], social adjustment problems among students 8–15 years of age [15], and poor social skills, inattention and depression among students 9–12 years of age [18]. Persistent victimization by peers is also associated with poor school performance among 9–10 year olds [19] and impaired social adjustment among 9–14 year old students [20]. There is some evidence that bullying victimization is more prevalent among psychiatric patients. Hansen, Hasselgard, Undheim and Indredavik [21] found that 19 % of young psychiatric outpatients aged 13–18 reported being bullied often or very often. Fosse and Holen [22] reported from a retrospective investigation that almost half (46 %) of the patients from an adult psychiatric outpatient clinic in Norway reported to have been bullied in childhood. Trotta et al. [23] found that adult patients with psychosis had approximately two-fold risk of reporting bullying victimization five or more years previously.

Social ecological theory [24] conceives human development as dynamic interrelations among various personal and environmental factors, such as neighborhood, home, school and society. Bullying could be understood within this framework as not only as the result of individual characteristics, but influenced by multiple relationships with i.e. peers, teachers and families [25]. Diathesis–stress model suggest that cognitive and biological vulnerabilities (i.e., diatheses) in interaction with environmental stressors are important in understanding the development of psychopathology [26]. Understood within these developmental models, involvement in bullying, as either a victim, perpetrator or both, can be seen as a negative life event, when mixed with the right vulnerabilities (i.e. cognitive, biological and social). This could contribute to the development of internalizing and externalizing psychopathology and impaired social relationships [25]. In early adolescence biological development (puberty and bodily changes) coincide with challenges in psychological (identity issues; cognitive development) and social development (increased autonomy from parents; increased social competence) possibly rendering some individuals vulnerable for external stressors, like being bullied.

Longitudinal studies suggest that problems following bullying involvement extend beyond mental health issues. Wolke, Copeland, Angold, and Costello [27] reported that those being exposed to bullying in adolescence, as either a bully or victim, had elevated risks for poverty, poor mental and physical health as well as poor social relationships in young adulthood. These risks were persistent even after controlling for family hardship and childhood psychiatric disorders. Takizawa, Maughan, and Arseneault [28] examined adult consequences of being bullied as a child in a prospective longitudinal study covering 50 years. They found that being bullied predicted poor psychosocial functioning in later years, psychological distress and poor physical health at ages 23 and 50, depression and poorer cognitive function in the later ages (45–50 years old). These findings suggest that bullying involvement, as a victim, perpetrator, or both, can impair later psychosocial functioning.

In light of the significant gaps in knowledge about the long-term outcomes following bullying involvement, we aim to examine the associations between bullying experiences at 14–15 years of age and mental health problems and psychosocial adjustment in young adulthood at 27 years of age in a community sample. We hypothesize that being involved in any type of bullying, either as victim, bully–victim or perpetrator, is associated with later internalizing and externalizing mental health problems, being bullied with more internalizing problems and thus being aggressive toward others more externalizing problems. Moreover, we predict that those being involved in bullying report more signs of poor psychosocial functioning, possibly strongly related to severe psychiatric problems than those non-involved. Using a longitudinal prospective follow-up of a representative community sample, we will differentiate among four types of bullying involvement to illuminate links with mental health and psychosocial functioning in young adulthood, including: (1) non-involved, (2) being bullied, (3) bully–victim, (4) aggressive toward others.

The following research aims were investigated in the present study:

1. How do experiences of being involved in bullying in adolescence affect later broad band internalizing and externalizing, and other more specific domains of mental health problems?
2. Do those being involved in bullying show lower levels of psychosocial functioning compared to those non-involved?
3. Do those being involved in bullying in adolescence receive more help for mental health problems and have more hospitalization compared to non-involved?

## Methods

### Sampling procedure

The Youth and Mental Health Study [29] is a longitudinal study conducted in Mid-Norway, aiming to address risk and protective factors in the development of mental health in adolescents aged 12–15 years. In 1998, a representative sample of 2813 students (98.5 % attending public schools) from 22 schools in two counties of Mid-Norway (South-and North-Trøndelag) was drawn with a probability according to size (proportional allocation) from a total population of 9292 children.

### Sample and assessment points

**Fig. 1:**
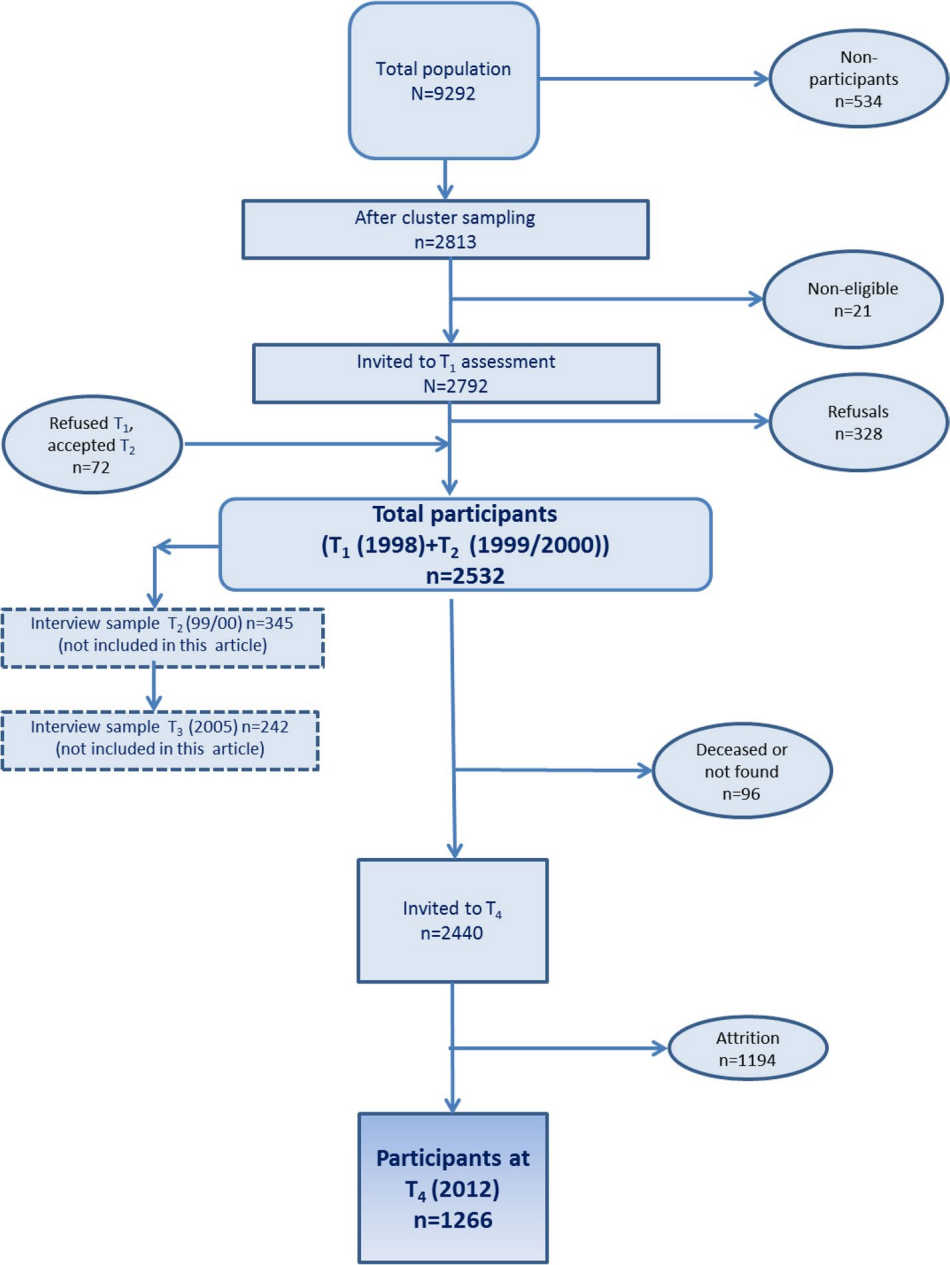
Schematic illustrating subject recruitment and attrition in the Youth and Mental Health Study wave 4 (T_4_)

Baseline data (T_1_) were collected in 1998 from 2464 adolescents, reflecting an 88.3 % response rate, with a mean age of 13.7 (SD 0.58, range 12.5–15.7) and 50.8 % girls, which were divided within four strata: (1) City of Trondheim (n = 484, 19.5 %), (2) Suburbs of Trondheim (n = 432, 17.5 %), (3) Coastal region (n = 405, 16.4 %), and (4) Inland region (n = 1143, 46.4 %) [29]. The sample was reassessed one year later (T_2_) with 2432 respondents at mean age 14.9 years (SD 0.6, range 13.7–17.0) and 50.4 % girls. Whereas 104 (4.3 %) from T_1_ did not participate at T_2_, 72 new participants who had changed their mind were added from the same schools. Data in these two waves were collected with questionnaires completed during two school hours. Individuals participating at T_1_ or T_2_ (N = 2532) were identified for a follow-up survey in young adulthood during the spring 2012 (this is referred to as T_4_ here because a portion of the T_2_ sample participated in an assessment at T_3_ unrelated to the objectives of the present study), about 12 years after T_2_ at a mean age of 27.2 years (SD 0.59, range 26.0–28.2). At T_4_, 92 were not eligible due to death (n = 13) or no identifiable home address (n = 79), resulting in that 2440 were invited to this follow-up investigation, of which 1266 (51.9 %) participated (56.7 % females) (see Fig. 1 for a detailed overview of the data collection). The data was collected electronically. All waves of data collection were approved by the Regional Committee for Medical Research Ethics in Mid-Norway.

### Measures in adolescence (T_1_ and T_2_)

*Report of being bullied* As part of a larger assessment, participants were asked if during the last 6 months, they had ever been (1) teased, (2) physical assaulted, or (3) frozen out of peer relationships at school or on the way to school. Responses was on a five-point scale (“never,” “1–2 times,” “about once a week,” “2–3 times a week,” and “more often”) [30].”

*Aggressive toward others* Four questions from the Youth Self Report (YSR) [31] addressed aggressive behavior: ‘‘I treat others badly,’’ ‘‘I physically attack people,’’ ‘‘I tease others a lot,’’ and ‘‘I threaten to hurt people’’. These are rated on a three-point scale (“not true,” “somewhat or sometimes true,’’ “very true or often true’’) for the previous 6 months were used. Because these items cannot differentiate aggression toward peers from other people (e.g., parent, teacher), this variable was termed aggressive toward others rather than bullying others.

*Classification of adolescent bullying involvement* From these items, participants’ involvement in bullying was classified as one of four types: *Being bullied* (n = 158, 66.5 % females): Reports of being bullied “about once a week” or more frequently, on one or more of the three items within the last 6 months at either T_1_ or T_2_. *Aggressive toward others* (n = 87, 42.5 % females): Reports of “very true or often true” within the past 6 months on at least one of the four YRS items indicating aggression toward others at either T_1_ or T_2_. *Bully*–*victim* (n = 39, 33.3 % females): Met classification of being bullied *and* being aggressive toward others, by the definitions above, within the last 6 months at either T_1_ or T_2._*Non*-*involved* (n = 982, 57.3 % females): Not classified as being bullied, aggressive toward others or bully–victim at either T_1_ or T_2_.

*The Youth Self Report (YSR)* [31], a 105-item self-rating of emotional, behavioral, and social problems in the last 6 months in children adolescents—was used to obtain background knowledge of baseline mental health at T_1_ with the global mental health measure YSR total problem scale. To prevent auto correlation, those items on the YSR total problem scale constituting the Aggressive toward others scale were removed in the controlled analyses.

*MFQ* The Mood and Feelings Questionnaire [32] was administered to measure depressive symptoms in more detail. MFQ is a 33-item questionnaire originally designed for children and adolescents ages 8–18 to report depressive symptoms as specified by the DSM-III Revised criteria [33], including affective, melancholic, vegetative, cognitive and suicidal symptoms. One item from the parent version was added. The individual is asked to report each symptom for the preceding 2 weeks using a three-point scale (0 = ‘‘not true’’, 1 = ‘‘sometimes true’’, and 2 = ‘‘true’’) resulting in the total summed scores range between 0 and 68. High scores represent high depressive symptom levels. In the present sample 3-week and 2-month test–retest reliabilities at T_1_ have been reported to be r = 0.84 and r = 0.80, respectively [34].

*Socio*-*economic status (SES)* was measured by adolescent report of mother’s and father’s occupation, in addition to an open question about what their parents did at work, which was classified according to the ISCO-88 [35] into professional leader, upper middle class, lower middle class, primary industry, and manual workers. Father’s occupation was used unless the adolescent lived with the mother only, in which case mother’s occupation was used.

### Outcome measures in young adulthood (T_4_)

The instruments administered at T_1_ and T_2_ were re-administered at T_4_ albeit with age appropriate adaptations.

*ASR*-*Mental health problems* at mean age 27.2 were assessed with the *ASR*—Adult Self-Report [36], which in the ASEBA system is the adult extension of the YSR addressing behavioral, emotional, and social problems, using the same response options. The ASR was selected because it has empirically based scales and has been shown to correlate with clinical diagnoses [31, 36–38]. The 120 problem items include broadband scales for Internalizing (anxious/depressed, withdrawn, somatic complaints), Externalizing (rule-breaking, aggressive behavior, intrusive), Attention Problems (concentration problems, disorganized behavior), and Critical Items (sum of 19 items). Critical items consist of specific atypical behavior which may be a concern in itself, regardless whether it reflects internalizing or externalizing problems. These types of behavior are termed as critical items, and contain “problems clinicians may be particularly concerned about”, for example “breaking things belonging to others”, “unhappy, sad or depressed”, “can’t get mind of certain thoughts” and “self-harming” [36]. A Total Problem score across all items can also be calculated.

*MFQ*—The Mood and Feelings Questionnaire [32] was re-administered at mean age 27.2 to give an concurrent measure on depressive symptoms.

*Psychosocial functioning* was measured with four questions related to state of mind [29]: One general question—“When you are worried or sad (having emotional or psychiatric problems) does it happen that you do not function as well as usually?” Responses were “True”, “Somewhat true” and “Not true”, with a timeframe within the last year. Three additional questions addressed different psychosocial functional areas: “Have you had to reduce/quit leisure activities due to a psychiatric problem for a while in the last year?”, “Have you been absent from school/work because of having emotional or psychiatric problems?” and “Have you had interpersonal problems caused by these problems during the last year? Response categories for these three questions were; “No,” “Less than 1 week,” “between 1 and 4 weeks,” or “more than 4 weeks”. Each question regarding psychosocial functioning was treated as dichotomous variables in the descriptives and ordinal variables in the logistic analyses.

*Received help for mental health problems* was measured by one question about receiving any help due to mental health problems during the last year, and one question asking about receiving any help due to mental health problems earlier in life. These questions had eleven response categories differentiating between types of help (i.e. psychologist or school health nurse). The eleven categories were dichotomized to a yes/no response. In addition a yes/no question were used asking about having ever been hospitalized because of mental health problems. This question was recoded based on a follow-up question about timeframe included, to distinguish hospitalization use after young adolescence (T_2_)_._

### Statistical analysis

One-way between-groups analyses of covariance were conducted to compare outcomes measured with continuous scales among the four bullying involvement groups. Participants’ gender and parent SES level were used as the covariates in this analysis. In additional analyses, the baseline mental health score was added as covariate. For the ordinal outcome variables, logistic regression analyses were used to compare the three bullying involvement groups with the noninvolved group as a reference. Ninety-five percent confidence intervals (CI) were computed. When performing six pairwise comparisons (Tables 1, 2) we used the Hochberg step-up procedure for multiplicity adjustment. The Hochberg procedure is generally recommended before the more conservative Bonferroni correction [39]. For the rest of the analyses, we have not adjusted for multiple hypothesis, as recommended by Rothman [40]. Two-sided p-values <0.05 are taken to indicate statistical significance. Due to multiple analyses, p-values between 0.01 and 0.05 should be interpreted with caution. In addition, cut-off points corresponding to the 90th percentile were used as indicators of possible mental health problems in the clinical range. This cut-off point is widely used in psychiatric epidemiology [41, 42]. Binary logistic regression analyses were used to test for associations between the different bullying groups and being a high-scorer (90th percentile) versus low-to-moderate-scorer on mental health outcomes, as well as receiving help for mental health problems. Analyses were performed in SPSS 21 and the Hochberg procedure was programmed in Excel.

**Table 1:** ANCOVA of ASR (Adult Self-Report) and MFQ (Mood and Feelings Questionnaire) scores for the different bullying involvement groups (Total N = 1266) adjusted for gender and parent SES-level

Outcomes	Non-involved (1) *M* (*SD*)	Being bullied (2) *M* (*SD*)	Bully–victim (3) *M* (*SD*)	Aggressive toward others (4) *M* (*SD*)	P value	Post-hoc comparison^a^
N	982	158	39	87		
ASR total problems (range 0–240)	30.34 (23.74)	39.61 (25.29)	46.41 (31.23)	39.68 (30.47)	<0.001	1 < 2, 3, 4
ASR externalizing problems (range 0–74)	6.55 (6.37)	8.69 (6.21)	10.33 (7.83)	9.46 (7.84)	<0.001	1 < 2, 3, 4
ASR internalizing problems (range 0–80)	10.82 (10.23)	14.87 (11.78)	16.83 (15.47)	13.75 (13.06)	<0.001	1 < 2, 3, 4
ASR attention problems (range 0–30)	5.40 (4.24)	6.30 (4.18)	8.21 (5.78)	6.63 (5.15)	<0.001	1 < 3
ASR critical items (range 0–38)	2.90 (3.11)	3.90 (3.09)	5.14 (4.19)	4.21 (4.35)	<0.001	1 < 2, 3, 4
MFQ depressive symptoms (0–68)	9.09 (11.25)	13.36 (13.62)	12.69 (13.16)	12.36 (13.86)	<0.001	1 < 2, 4

^a^Hochberg’s step-up correction

**Table 2:** ANCOVA of ASR (Adult Self-Report) and MFQ (Mood and Feelings Questionnaire) scores for the different bullying involvement groups (Total N = 1266) adjusted for gender and parent SES-level and baseline mental health score

Outcomes	Non-involved (1) *M* (*SD*)	Being bullied (2) *M* (*SD*)	Bully–victim (3) *M* (*SD*)	Aggressive toward others (4) *M* (*SD*)	P value	Post-hoc comparison^a^
N	982	158	39	87		
ASR total problems (range 0–240)	30.27 (23.70)	39.70 (25.34)	46.41 (31.23)	39.68 (30.47)	0.057	N.S
ASR externalizing problems (range 0–74)	6.53 (6.37)	8.68 (6.23)	10.38 (7.83)	9.46 (7.84)	0.060	N.S
ASR internalizing problems (range 0–80)	10.79 (10.22)	14.94 (11.79)	16.84 (15.47)	13.75 (13.06)	0.055	N.S
ASR attention problems (range 0–30)	5.39 (4.24)	6.30 (4.19)	8.22 (5.78)	6.63 (5.15)	0.239	N.S
ASR critical items (range 0–38)	2.89 (3.10)	3.91 (3.10)	5.14 (4.19)	4.21 (4.35)	0.008	N.S
MFQ depressive symptoms (0–68)	9.05 (11.13)	13.36 (13.61)	12.68 (13.16)	12.36 (13.86)	<0.001	1 < 2

Baseline mental health score for ASR(T_4_); YSR total problem score (T_1_), baseline mental health score for MFQ(T_4_); baseline MFQ score (T_1_)

^a^Hochberg’s step-up correction

## Results

### Sample characteristics

The total study sample (N = 1266) comprised 56.7 % females. The prevalence of any bullying involvement in adolescence at T_1_ or T_2_ was 22.4 % (n = 284). Among these was 12.5 % (n = 158) being bullied, 6.9 % (n = 87) being aggressive toward others, and 3.1 % (n = 39) being a bully–victim, leaving the prevalence of non-involvement in any of the bullying groups at 77.5 % (n = 982).

### Attrition analysis

The responders at T_4_ were compared with the non-responders on gender, parental SES, ethnicity and bullying classification assessed at T_1_/T_2_. The responders were characterized by more females than non-responders (56.9 vs. 44.4 %, χ^2^ (1) = 39.44, p < 0.001) and fewer with non-Norwegian ethnicity (1.7 vs. 3.6 %, χ^2^ (1) = 8.79, p = 0.003.). There were also parental SES differences between responders and non-responders (χ^2^ (4) = 27.20, p < 0.001). Subsequent Chi square goodness of fit tests showed that upper middle class was overrepresented among responders (33.6 vs. 25.5 %, χ^2^ (1) = 17.19, p < 0.001) whereas workers were underrepresented (34.1 vs. 41.8 %, χ^2^ (1) = 5.93, p < 0.015). In the total sample, attrition rate for T_4_ was 48.1 %. Specifically among the groups involved in bullying the attrition rate for T_4_ was: being bullied (47.3 %), bully–victim (40.0 %), and aggressive toward others (56.7 %). Chi square tests for each sub-group involved in bullying showed no significant difference in proportional rates between those participating at T_4_ versus those not.

### Young adult outcomes associated with bullying involvement

Table 1 shows the mean scores for the different bullying involvement groups for ASR (Adult Self Report) broadband Total, Externalizing, Internalizing, Attention and Critical Problems scales, as well as MFQ depressive symptoms. As shown in Table 1, after controlling for gender and parents SES level, ANCOVAs indicated there were differences among the bullying involvement groups on ASR total-, externalizing- and internalizing-problems and the critical problems scales (all p < 0.001). Post hoc comparisons showed that being bullied, bully–victim, and aggressive toward others had significantly higher problem levels than non-involved. ASR attention problems were also significantly different, with post hoc comparisons showing that only bully–victims had significantly higher scores than non-involved. Moreover, depression symptom scores as measured on the MFQ (Mood and Feelings Questionnaire) were significantly different, with post hoc comparisons showing that being bullied and those being aggressive toward others had significantly higher scores than non-involved. However, post hoc comparisons showed only differences compared with the non-involved and no differences on any of the measurements between the groups involved in bullying occurred. After adjusting for baseline mental health as seen in Table 2, only depressive symptoms among those being bullied compared to non-involved, remained significant.

Comparing psychosocial functioning outcomes as descriptives (as shown in Table 3) and with ordinal logistic regressions (shown in Table 4), controlling for gender and parent SES, indicated that those being bullied had a higher risk of reporting reduced general functioning (*OR* 1.69, 95 % CI 1.21–2.36, p < 0.002) during the last year compared to the reference group of non-involved. Both those being bullied and aggressive toward others more often reported reduced leisure activities in comparison with non-involved (*OR* 1.76, 95 % CI 1.06–2.94, p = 0.03 and *OR* 2.53, 95 % CI 1.35–2.76, p = 0.004, respectively).

**Table 3:** Dichotomized descriptive psychosocial and mental health characteristics as young adults (T_4_) in different bullying involved groups in adolescence (Total N = 1266)

Variables	NNon-involved (*n* = 982) [%(n)]	Being bullied (*n* = 158) [%(n)]	Bully–victim (n = 39) [%(n)]	Aggressive toward others (n = 87) [%(n)]	Total sample (n = 1266) [%(n)]
Reduced functioning (Y/N)	40.6 (371)	55.4 (82)	44.4 (16)	44.7 (34)	39.7 (503)
Reduced leisure activities (Y/N)	6.3 (58)	10.1 (15)	2.8 (1)	13.2 (10)	6.6 (84)
Absence from school/work (Y/N)	7.9 (72)	8.8 (13)	13.9 (5)	10.5 (8)	7.7 (98)
Affected interpersonal relations (Y/N)	8.2 (75)	10.8 (16)	13.9 (5)	7.9 (6)	8.7 (102)
ASR total problem—high scorers (Y/N)^a^	8.1 (79)	17.1 (8)	20.5 (8)	19.5 (17)	8.8 (112)
ASR externalizing—high scorers (Y/N)^a^	9.3 (91)	13.9 (22)	23.1 (9)	20.7 (18)	11.1 (140)
ASR internalizing—high scorers (Y/N)^a^	8.2 (80)	16.5 (26)	23.1 (9)	18.4 (16)	10.3 (131)
ASR attention—high scorers (Y/N)^a^	9.9 (97)	12.7 (17)	23.1 (9)	19.5 (17)	11.1 (140)
ASR critical items—high scorers (Y/N)^a^	9.1 (89)	17.1 (27)	33.3 (13)	18.4 (16)	11.5 (145)
MFQ depressive symptoms—high scorers (Y/N)^a^	8.8 (86)	16.5 (26)	12.8 (5)	16.1 (14)	10.3 (131)
Received mental health help last year (Y/N)	28.2 (277)	39.2 (62)	28.2 (11)	35.6 (31)	30.1 (381)
Received mental health help earlier in life (Y/N)	33.1 (325)	48.7 (77)	38.5 (15)	41.4 (36)	35.8 (453)
Psychiatric hospitalization since T_2_ (Y/N)	1.5 (15)	5.1 (8)	7.7 (3)	9.2 (8)	2.7 (34)

^a^Dichotomized being a high-scorer (90th percentile) versus low-to-moderate-scorer on mental health outcomes in young adulthood

**Table 4:** Adjusted odds ratios from ordinal logistic regression comparing the different bullying involved groups with the non-involved group in adolescence with the outcome of psychosocial functioning in young adulthood (Total N = 1266)

Non-involved vs.	Being bullied (n = 158)			Bully–victim (n = 39)			Aggressive toward others (n = 87)		
	OR	95 CI	P value	OR	95 CI	P value	OR	95 CI	P value
Reduced functioning	1.69	1.21–2.36	0.002	1.30	0.66–2.55	0.447	1.39	0.88–2.18	0.161
Reduced leisure activities	1.76	1.06–2.94	0.029	0.39	0.05–2.86	0.353	2.53	1.35–4.76	0.004
Absence from school/work	1.31	0.85–2.03	0.224	1.91	0.85–4.28	0.117	1.25	0.68–2.29	0.475
Affected interpersonal relations	1.27	0.82–1.95	0.285	0.93	0.36–2.41	0.879	0.93	0.49–1.76	0.819

Adjustments made for gender and parent SES. Range on all variables = 1–4, with higher scores indicating negative outcomes

Using the 90th percentile as a cut-off value for being a high scorer on the ASR and MFQ scale, a series of univariate logistic regressions (see Table 5) controlled for gender and parent-SES were performed. The results showed that being bullied, bully–victims and aggressive toward others had an increased risk of being above the 90th percentile on the ASR total problem scale (all p values <0.01), on the ASR externalizing scale (p < 0.05, p < 0.05 and p < 0.01, respectively), and having an increased risk of reporting high scores on ASR internalizing problems compared to non-involved (all p-values ≤0.01). Further, being a high scorer on the ASR attention problem scale differed between bully–victims and those aggressive toward others compared to non-involved (both tests, p = 0.004). Moreover, those involved in bullying compared to non-involved, had increased risk of a being high-scorer on ASR critical problems (all p-values p < 0.01). However, when adjusting for baseline mental health in addition to gender and parent-SES (Table 6) results showed that only those being bullied and aggressive toward others had an increased risk of being above the 90th percentile on the ASR total problem scale [both p < 0.05) and ASR internalizing scale (p = 0.017 and p = 0.014, respectively)]. While those being bullied and bully–victims in addition had an increased risk of scoring above the 90th percentile on the ASR critical items (p = 0.036 and p = 0.003, respectively). Lastly, those being bullied and those aggressive toward others had in the analyses controlling for gender and parents SES level an increased risk of being a high-scorer on the MFQ, the depressive symptom scale, compared to non-involved (p = 0.009 and p = 0.014, respectively), while when adjusting for MFQ levels at T1 none of the associations remained significant.

**Table 5:** Adjusted odds ratios (95 % CI) from binary logistic regression analyses comparing the different bullying groups in adolescence and being a high-scorer (90th percentile) versus low-to-moderate-scorer on mental health outcomes in young adulthood (Total N = 1266)

Non-involved (n = 982) vs.	Being bullied (n = 158)			Bully–victim (n = 39)			Aggressive toward others (n = 87)		
	OR	95 CI	P value	OR	95 CI	P value	OR	95 CI	P value
ASR total problems	2.42	1.48–3.94	<0.001	3.36	1.41–8.04	0.006	3.28	1.82–5.93	<0.001
ASR externalizing problems	1.68	1.02–2.79	0.044	2.61	1.15–5.92	0.022	2.49	1.41–4.40	0.002
ASR internalizing problems	2.33	1.42–3.80	0.001	4.25	1.83–9.87	0.001	3.17	1.73–5.82	<0.001
ASR attention problems	1.28	0.74–2.19	0.379	3.24	1.47–7.15	0.004	2.37	1.32–4.21	0.004
ASR critical items	2.04	1.27–3.30	0.003	5.06	2.4–10.53	<0.001	2.32	1.29–4.19	0.005
MFQ depressive symptoms	1.92	1.18–3.13	0.009	1.89	0.71–5.05	0.206	2.19	1.17–4.10	0.014

Adjustments made for gender and parent SES

**Table 6:** Adjusted odds ratios (95 % CI) from binary logistic regression analyses comparing the different bullying groups in adolescence and being a high-scorer (90th percentile) versus low-to-moderate-scorer on mental health outcomes in young adulthood (Total N = 1266)

Non-involved (n = 982) vs.	Being bullied (n = 158)			Bully–victim (n = 39)			Aggressive toward others (n = 87)		
	OR	95 CI	P value	OR	95 CI	P value	OR	95 CI	P value
ASR total problems	1.87	1.12–3.11	0.017	1.75	0.69–4.44	0.238	2.17	1.16–4.07	0.016
ASR externalizing problems	1.34	0.79–2.26	0.274	1.55	0.65–3.70	0.323	1.78	0.97–3.24	0.061
ASR internalizing problems	1.87	1.12–3.10	0.017	2.43	0.99–6.00	0.053	2.22	1.17–4.21	0.014
ASR attention problems	1.06	0.61–1.85	0.843	2.07	0.90–4.81	0.089	1.76	0.95–3.24	0.071
ASR critical items	1.70	1.03–2.79	0.036	3.31	1.52–7.20	0.003	1.73	0.93–3.23	0.083
MFQ depressive symptoms	1.61	0.97–2.68	0.064	1.20	0.43–3.39	0.726	1.62	0.84–3.14	0.154

Adjustments made for gender and parent SES and and baseline mental health score

Baseline mental health score for ASR(T4); YSR total problem score (T_1_), baseline mental health score for MFQ(T4); baseline MFQ score (T_1_)

A sensitivity analysis was performed to assess if a 90 % cut-off was reasonable, assessing different threshold levels on the actual outcome (85th, 90th, 95th percentiles). This analysis showed in terms of significance, similar results for the 85th and 90th percentile (as shown in the current Table 5).

As shown in Table 7, all groups involved in bullying in adolescence had four- to eight-fold higher risk of being hospitalized due to mental health problems since T_2_ compared to non-involved. Those being bullied in adolescence reported as young adults’ 63 % higher risk of receiving any help due to mental health problems during the last year, and 94 % increased risk of having received any help earlier in life, compared to non-involved. However, the other bullying involved groups were no different from non-involved.

**Table 7:** Adjusted odds ratios (95 % CI) from binary logistic regression adjusted with gender and parent—SES comparing the different bullying involved groups with the non-involved group in adolescence on reported received mental health help and inpatient hospitalization since T_2_ as young adults due to mental health problems (Total N = 1266)

Non-involved vs.	Being bullied (n = 158)			Bully–victim (n = 39)			Aggressive toward others (n = 87)		
	OR	95 CI	*P* value	OR	95 CI	P-value	OR	95 CI	P-value
Received mental health help last year	1.63	1.15–2.33	0.007	1.18	0.57–2.43	0.656	1.51	0.95–2.40	0.084
Received mental health help earlier in life	1.94	1.38–2.74	<0.001	1.41	0.71–2.79	0.328	1.57	99–2.46	0.051
Psychiatric hospitalization since T2	3.94	1.58–9.82	0.003	8.13	2.14–30.88	0.002	8.63	3.84–22.00	<0.001

Adjustments made for gender and parent SES

## Discussion

The aim was to examine associations between bullying experiences at 14–15 years and mental health problems and psychosocial functioning in young adulthood at 27 years. In the results, controlling for gender and parents SES level, all groups involved in bullying in adolescence reported higher levels of mental health problems in adulthood, including broadband total, externalizing and internalizing problems, compared to the group who reported no such experience. Moreover, bully–victims reported significantly higher attention problems in adulthood compared with non-involved. Also those being bullied and those aggressive toward others reported more depressive symptoms as measured by the MFQ. However, when adjusting for baseline mental health problems, only those being bullied retained a significant result on depressive problems. Results controlling for gender and parents SES level and in addition adjusted for baseline mental health showed that being involved in bullying as being bullied, bully–victim or aggressive toward others increased the odds of reporting a higher odds of being a high scorer on problems scales across the range of mental health outcomes compared to non-involved. These findings suggest that not only does involvement in bullying in adolescence act as a risk factor across the mental health spectrum in young adulthood, but also that there is a disproportional shift toward the top end of that range. This suggests that involvement in bullying contribute to vulnerability to mental health problems in young adulthood, and should be seen as a harmful public health risk.

Research has previously established that bullying may be a risk factor for later depression in adolescence [14] and young adulthood [1]. Regarding later depressive problems the results in the present study show, when adjusting for baseline depressive symptom levels, that those being bullied report significantly more depression symptoms than those non-involved in young adulthood. The finding that those being bullied specifically have a depression outcome is a strong argument that victims experience long-term impairment in the long run by their experience. However, when assessing high scorers of mental health problems versus low-to middle scorers, in controlled analyses, both victims and those aggressive toward others show high levels of internalizing problems, however not on depressive symptoms. Internalizing problems are not only composed of depression but also contain components such as anxiety, fear and withdrawal from social contacts. Starr and Davila [43] found that while there were many features common to both depression and general anxiety, social anxiety has shown to have a greater correlation with peer variables (e.g., social competence, communication in friendships). Bullying has been characterized as a peer relationship problem [44]. Involvement in bullying both as victim and aggressor might be an anxiety provoking experience, which could leave longstanding marks. It is thus particularly important to understand the development of anxiety from adolescence to young adulthood among those who are involved in bullying.

A possible link between an aggressive trait and depression and other internalizing symptoms, may be mediated through relational problems i.e. domestic problems with depression and anxiety as a possible outcome. Surprisingly, bully–victims did not report significantly elevated depressive symptoms, which might be the result of the small size of this group in this study. On the other hand, it could be that bully–victims have another reaction pattern than the other bullying involvement groups. Given that bully–victims display more adjustment problems among all children involved in bullying [45], it could in the long run turn into more externalizing problem tendencies such as rule-breaking behavior or a tendency to reactive aggression or other internalizing problems such as anxiety [46]. This was in part confirmed by our findings, when high-scorers compared to low-to-moderate scores with non-involved as baseline, bully–victims had higher odds than the other involved groups in bullying on internalizing and critical problems in both analyses adjusted and unadjusted for baseline mental health.

Critical problems may indicate a clinical concern and behavior that deviate markedly from more typical problem behavior, such as breaking things belonging to others or self-harm. Those involved in bullying, again regardless of type of experience, reported more critical problems than those non-involved, Also, a higher proportion of high-scorers on critical problems were evident in the groups involved in bullying than those non-involved. However, when adjusting for baseline mental health these finding were retained for those being bullied and bully–victims only. In line with the externalizing and internalizing findings, those involved in bullying in adolescence seems to be at risk for significant psychiatric morbidity in young adulthood and victims being strongest affected. This finding was confirmed in that all those involved in bullying in adolescence had higher risk of having a history of hospitalization due to mental health problems in young adulthood.

We hypothesized that adolescent bullying involvement would predict poorer psychosocial functioning in young adulthood including reduced leisure activities, more absence from school/work, and affected interpersonal relations. Results partly confirmed this in that those being bullied reported reduced general psychosocial functioning as young adults compared to those non-involved and both those being bullied and aggressive toward others reported reduced leisure activities. A general reduced psychosocial functioning in young adulthood could be caused by social vulnerability and trust issues caused by past bullying experiences [47]. Further, the results could be mediated by, the higher levels of depression symptoms reported among those being bullied and being aggressive toward others in adolescence. This could imply that being depressed could negatively impact the level of leisure activities.

The 14 year length of time between the first measurement of bullying-involvement and measurement of mental health and psychosocial functioning adverse outcomes might indicate a long lasting effect on the individual. In regard to using the health system as young adults, only the group being bullied was significantly more likely than non-involved to have been receiving mental health services earlier in life and in the last year. Those being bullied appear to be at higher risk of currently using mental health services even if the bullying exposure happened over a decade in the past. However, all groups involved in bullying had increased risk of mental health hospitalization since T_2_: those being bullied reported a four-fold higher risk and both bully–victims and those aggressive toward others reported an eight-fold higher risk than their non-involved peers. This is an important marker of severity of mental health problems in adulthood which adds to previous findings that adverse mental health outcomes associated with involvement in childhood bullying are also exhibited into adulthood [1, 5, 6, 48].

### Strengths and limitations

The longitudinal perspective in this study captures an important developmental transition from dependent childhood to early adulthood when considerable, if not complete, independence is expected [49]. It provides stronger evidence how bullying involvement can exhibit effects over a decade later than previous studies have been able to do relying on clinical samples or retrospective reports.

Whereas the sample followed in the present study is representative of the community from the region of Mid-Norway, it is not a national representative sample. All data were based upon self-report. Respondents might for various reasons give inaccurate or biased information, such as social conforming responses. However, when confidentiality and anonymity are granted as in this study, self-report typically has high reliability and validity [50].

Social ecological theory [24] and the diathesis-stress model [26] have been used to explain how stressful life experiences such as bullying interact with biology to influence the development of mental health problems. Although difficult to assess in a non-experimental design, it is probable that these relations are transactional, with preexisting mental health problems also putting individuals at greater risk for stressful life experiences and vice versa stressful life experiences put the individual at risk for mental health problems. Young adolescents who are involved in bullying may have characteristics that make them more vulnerable for mental health problems, i.e. those aggressive toward others could initially have more externalizing problems and those being bullied could have more introvert, non-assertive behavior. Our analyses controlling for baseline mental health does only partly address the suspected bidirectional relationship between mental health problems and bullying involvement as temporal priority is the foremost criterion for testing causal effects.

Bully–victims were a relatively small group in our sample with large variations in outcomes. Many of the findings in the unadjusted analyses disappeared when the analyses were controlled for baseline mental health at T1. This might be caused by a small group size, with differences not reaching significance levels and because long term outcomes in this group was strongly related to mental health problems already apparent already at the age of 14. Future research with larger samples should explore bully–victims in particular with regard to mental health and psychosocial functioning.

Another limitation of our study is the measure of “aggressive toward others” represented by four questions. These do not specify forms of bullying nor exclusively toward peers. Importantly, relational aggression, such as spreading rumors or excluding individuals from social groups, which has been found to be more characteristic of female bullies, is not addressed in this measure [51]. Therefore, the group “aggressive toward others” may be over-represented in our sample by male bullies, who more often engage in this type of bullying.

A limitation to the assessment of bullying involvement was that it was measured only in the two last years of middle school. Ideally one would have liked to follow the adolescents up after each school year within middle school and possibly over to the first years of high-school, to get an even better understanding of the developmental trajectories of involvement in bullying. This was not done, due to economic constraints. However, several studies has shown that involvement in bullying peaks in the end of middle school, followed by a decline as high school precedes [52,53].

Using post hoc thresholds of item scales has its limitations. The very best option to delineate those in the normal range versus clinical range would have been to observe people with different levels for a sustained period, and identify a threshold beyond which people start feeling the burden in some sense. However, this is an extremely complex procedure involving consultation from experts, and is beyond the scope of our research material. A sensitivity analysis using different thresholds levels showed that the 90th percentile was robust as a cut-off point.

Although the response rate was excellent at both T_1_ and T_2_, it was modest at T_4_, although this was 14 years after the first wave and a drop in response rate certainly would be expected. In our study, we obtained follow-up data from 1266 individuals. We regard the follow up rate (51.9 %) as neither particularly low nor high, compared to what is often seen in observational studies over this duration. Moderate response rates can be a problem if the sample is systematically different from the population it is supposed to represent. Attrition analyses showed that even if there were small differences between the responders and non-responders regarding gender, parent SES and ethnicity, there were no differences in attrition associated with bullying involvement. Moreover, this sample is large and heterogeneous and constitutes variation in gender and geographical and sociocultural markers, indicating that the sample is valid and likely generalizable to the target population.

## Conclusion

The present study has some main findings. Firstly, all groups involved in bullying in young adolescence had adverse mental health outcomes at 27 years compared to non-involved. Specifically, those being bullied and those being aggressive toward others showed reduced mental health in adulthood compared with non-involved and both groups showed reduced leisure activities than their non-involved peers. Those being bullied were strongly affected, especially regarding increased total sum of depressive symptoms and high levels of total, internalizing and critical symptoms, increased risk of having received help for mental health problems and reduced functioning because of a psychiatric problem. While those being aggressive toward others showed high levels of total and internalizing symptoms. Both those being bullied and bully–victims showed an increased risk of high levels of critical symptoms. Lastly, those involved in bullying as being bullied, bully–victim and aggressive toward others, had increased risk of psychiatric hospitalization because of mental health problems since T_2_, compared to those who had no bullying experience. These findings reinforce implementing zero-tolerance policies toward bullying in schools and provide a strong argument that prevention of bullying should start as early as possible. In the clinical practice questions about past bullying experiences should not be missed as it seems to be highly relevant to mental health outcomes in young adulthood.
